# Efficacy and safety of naphlimostat mesylate versus sodium citrate in CRRT for EICU patients with coagulation dysfunction: A STROBE-compliant observational study

**DOI:** 10.1097/MD.0000000000044932

**Published:** 2025-10-03

**Authors:** Binglin Song, Chun Liu, Kangrui Fu

**Affiliations:** a Clinical Medical College, North Sichuan Medical College, Nanchong, Sichuan, China; b Dazhou Central Hospital, Dazhou, Sichuan, China.

**Keywords:** coagulation dysfunction, continuous renal replacement therapy, in vitro anticoagulation, naphlimostat mesylate

## Abstract

This study aimed to evaluate the safety of naphlimostat mesylate (NM) as an anticoagulant in vitro for emergency intensive care unit patients with coagulation dysfunction undergoing continuous renal replacement therapy (CRRT). Patients requiring CRRT and presenting with coagulation dysfunction, admitted to Dazhou Central Hospital’s emergency intensive care unit in Sichuan Province between September 2021 and January 2023, were included. Patients were randomly assigned to either the NM group (experimental) or the sodium citrate group (control). Data on patient demographics, changes in coagulation factors, hemoglobin, platelets, inflammatory markers, and electrolytes during treatment were collected for both groups. A total of 89 patients participated, with 49 in the NM group and 40 in the sodium citrate group. Complete data were available for 39 patients in the NM group and 36 in the sodium citrate group. Data on filter usage, alterations in coagulation factors, hemoglobin, platelets, inflammatory markers, and electrolytes were collected throughout the treatment period. A total of 117 filters were used across both groups. The median filter duration was 25.5 (range 16.0–43.5) hours in the NM group and 28.5 (range 18.0–46.5) hours in the sodium citrate group. There was no statistically significant difference in daily filter usage between the groups (1.16 ± 0.76 vs 1.00 ± 0.55, *P* > .05). NM demonstrated comparable anticoagulant efficacy to sodium citrate. Posttreatment, there were no significant differences between groups in prothrombin time (PT) (35.95 ± 33.14 vs 23.71 ± 19.37), activated partial thromboplastin time (55.65 ± 20.82 vs 55.65 ± 20.82), international normalized ratio (3.05 ± 2.83 vs 4.91 ± 10.60), hemoglobin (82.10 ± 15.01 vs 93.10 ± 26.86), platelet count (66.70 ± 59.87 vs 70.10 ± 48.85), serum sodium (138.80 ± 5.92 vs 142.00 ± 3.59), serum potassium (4.00 ± 0.52 vs 3.96 ± 0.61), serum calcium (1.13 ± 0.19 vs 0.99 ± 0.28), white blood cell count (10.82 ± 5.81 vs 12.54 ± 6.32), and neutrophil count (8.73 ± 5.05 vs 10.89 ± 5.94) levels (all *P* > .05). NM is a safe and effective extracorporeal anticoagulant, demonstrating comparable anticoagulant efficacy to sodium citrate without increasing the risk of bleeding in patients with coagulation abnormalities undergoing CRRT.

## 1. Background

Acute and critically ill patients are at higher risk of developing multiple organ dysfunction syndrome, including acute kidney injury (AKI). Approximately 15% to 20% of these patients require continuous renal replacement therapy (CRRT).^[1]^ However, many patients often experience coagulation dysfunction in addition to other conditions.^[2]^ Particularly in cases of severe bodily injury, various factors such as activation of the coagulation system, deficiency of anticoagulants, suppression of fibrinolysis, and other variables contribute to coagulation dysfunction.^[3,4]^ Extracorporeal coagulation is common during CRRT, and anticoagulation is crucial to ensure the proper functioning of CRRT. Currently, heparin is frequently used as an anticoagulant; however, it is associated with risks such as bleeding and thrombocytopenia.^[5,6]^ Expert consensus and guidelines recommend the use of sodium citrate as an alternative anticoagulant for patients with coagulation abnormalities. It is important to note that sodium citrate may lead to internal environmental disturbances.^[7]^ Severe liver failure is listed as a contraindication for sodium citrate anticoagulation therapy due to the potential for sodium citrate accumulation and subsequent metabolic complications.^[8]^ Consequently, there is a critical need to explore alternative in vitro anticoagulants that are both safer and more effective. Research has highlighted naphlimostat mesylate (NM) as an anticoagulant used in 84.3% of critically ill Japanese patients undergoing blood purification, a significantly higher percentage compared with heparin (11.5%).^[7,9]^ NM, a synthetic serine protease inhibitor, offers multiple advantages including broad-spectrum anticoagulation, versatile metabolism, and a short half-life. Preliminary studies have shown promising results of NM as an anticoagulant in CRRT for patients with sepsis and AKI, demonstrating good safety and efficacy. However, these studies are limited by small sample sizes, single-center designs, and lack of comparative studies with other anticoagulants such as sodium citrate.^[10,11]^ Currently, there is a lack of comparative clinical studies investigating the use of NM and sodium citrate in CRRT for patients with coagulation dysfunction. Therefore, this study aims to evaluate the safety and efficacy of these 2 anticoagulants in the CRRT setting, with the goal of providing evidence-based guidance for clinical decision-making.

## 2. Data and methods

### 2.1. Study subjects

This randomized controlled study enrolled a total of 89 patients requiring CRRT at Dazhou emergency intensive care unit between September 2021 and January 2023. The cohort consisted of 49 males and 40 females with a mean age of 58.8 years. Patients were randomly assigned to either the NM group (experimental group) (manufactured by Jiangsu Durui Pharmaceutical Co., Ltd.) or the sodium citrate group (control group). In the NM group, there were 49 patients (21 males, 18 females), while the sodium citrate group included 40 patients (19 males, 17 females), with valid data obtained from 36 and 36 patients, respectively. General conditions and changes in coagulation factors, hemoglobin levels, inflammatory markers, electrolytes, and other parameters during treatment were recorded for both groups. This study was approved by the Ethics Committee of Dazhou Central Hospital (Ethics number: 2021 review (059)).

### 2.2. Inclusion criteria

Inclusion criteria are as follows: first, age (18–70); second, meets the Chinese expert consensus on standardized assessment of coagulopathy in severe patients^[12]^; third, CRRT treatment, CRRT treatment indication: AKI greater than or equal to stage 2; pH < 7.1; anuria/oliguria (urine volume <50 mL/12 h); severe electrolyte metabolic imbalance (sodium ion >160 mmol/L or <115 mmol/L, potassium ion >5.5 mmol/L); risk of pulmonary edema or acute respiratory distress syndrome; coagulation disorders require large input of blood products; and CRRT to regulate internal environment balance^[13]^; fourth, Before CRRT (activated partial thromboplastin time [APTT]) >37 or <23 seconds; international standardized ratio (INR) >1.3 or <0.8 or 24 hours; fifth, patients were required to voluntarily participate in this clinical trial and sign an informed consent.

### 2.3. Exclusion criteria

Exclusion criteria are as follows: unmatched treatment (e.g., mental illness, role behavior); required anticoagulation for reasons other than CRRT; participated in other clinical trials during nearly 1 month; allergic constitution; pregnant or lactating women; incomplete medical records of control patients; and other conditions considered by the investigator for enrollment.

### 2.4. Treatment regimen

All patients underwent continuous veno-venous hemodiafiltration using a Japanese Fresenius machine, specifically the Prismaflex ST150 system, equipped with an AN69 membrane 67 hollow fiber filter (1.5 m^2^ membrane area). The blood flow rate ranged from 150 to 200 mL/min. A commercial standard bicarbonate replacement solution (from Chengdu Qingshan Likang Pharmaceutical Co., Ltd.) was used, with a prescribed dose of 30 to 35 mL/(kg/h).

Patients in the NM group: prior to treatment, use 1000 mL of saline containing 5000 U of heparin to flush the circuit and filter; preload with 1000 mL of saline; the initial loading dose prior to treatment is 0.4 mg/kg; after treatment initiation, maintain a dose of 0.4 mg/kg/h.

Patients in the sodium citrate group (200 mL; 8 g, with the main component being sodium citrate): first, sodium citrate was connected to the arterial end of the hemofilter line through an infusion pump, and the anticoagulation target was to maintain the free calcium level at 0.2 to 0.4 mmol/L after controlling the filter. Second, by collecting arterial blood gas, checking the concentration of calcium ions, and supplementing the calcium-containing replacement solution to the venous end of the blood filter line, the purpose is to keep the free calcium in the blood back to the body at normal levels as far as possible.

### 2.5. Observational indicators

Collect the basic clinical data of the 2 groups; observed and recorded the overall filter life, laboratory results before, during and after treatment, including APTT, PT, INR, serum sodium, serum potassium, serum calcium, and inflammatory factors; observe and record bleeding events and adverse reactions in both groups.

### 2.6. Statistical methods

All data were subjected to statistical analysis using SPSS 27.0 software (IBM Corp., Armonk). Normally distributed data were analyzed using the Student *t* test, and results are presented as mean ± standard deviation ($\bar{x}\pm s$). Non-normally distributed data were analyzed using the Mann–Whitney *U* test, and results are presented as median with interquartile range (*M*(Q1, Q3)). The lifetimes of the 2 filter types were visualized using box plots and Kaplan–Meier curves. A 2-sided test was employed, with a significance level set at α = 0.05. Differences were considered statistically significant when *P* < .05.

## 3. Results

**Figure 1.**
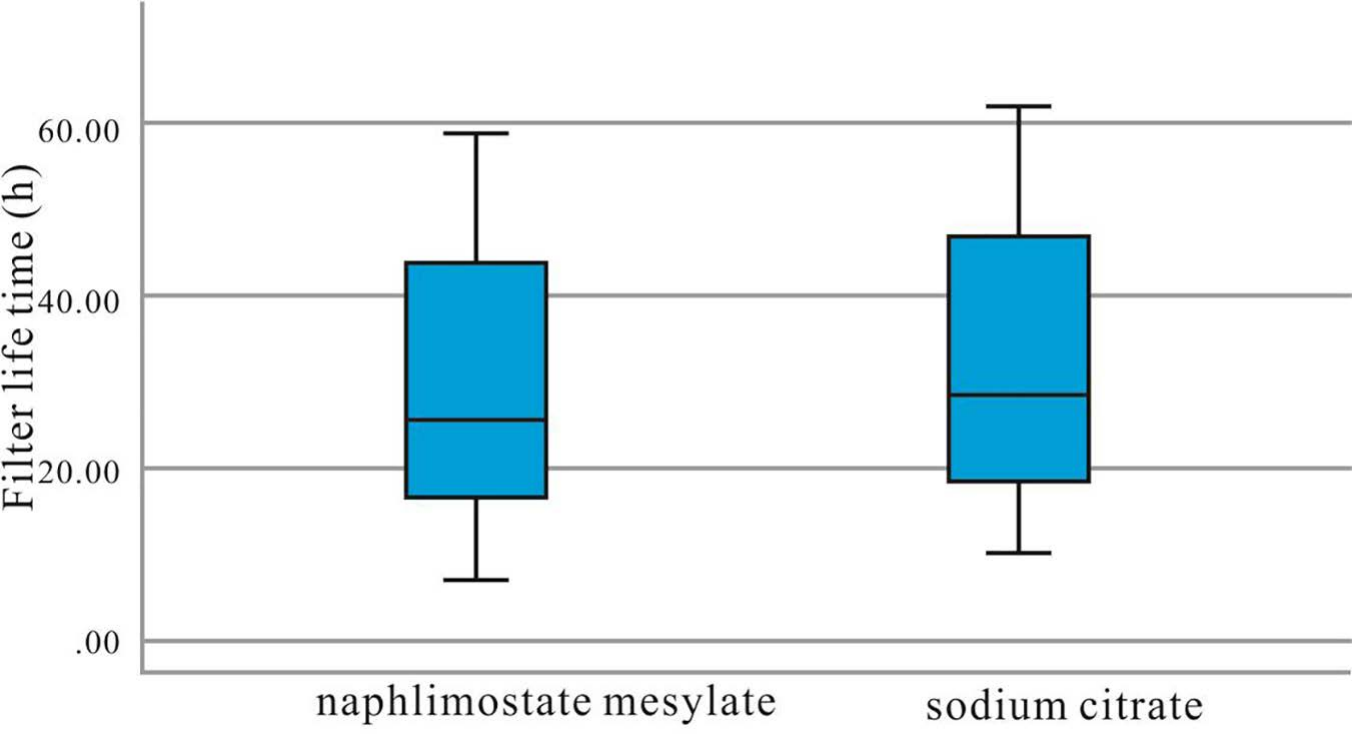
Box plot of CRRT filter life (*P* > .05). CRRT = continuous renal replacement therapy.

**Figure 2.**
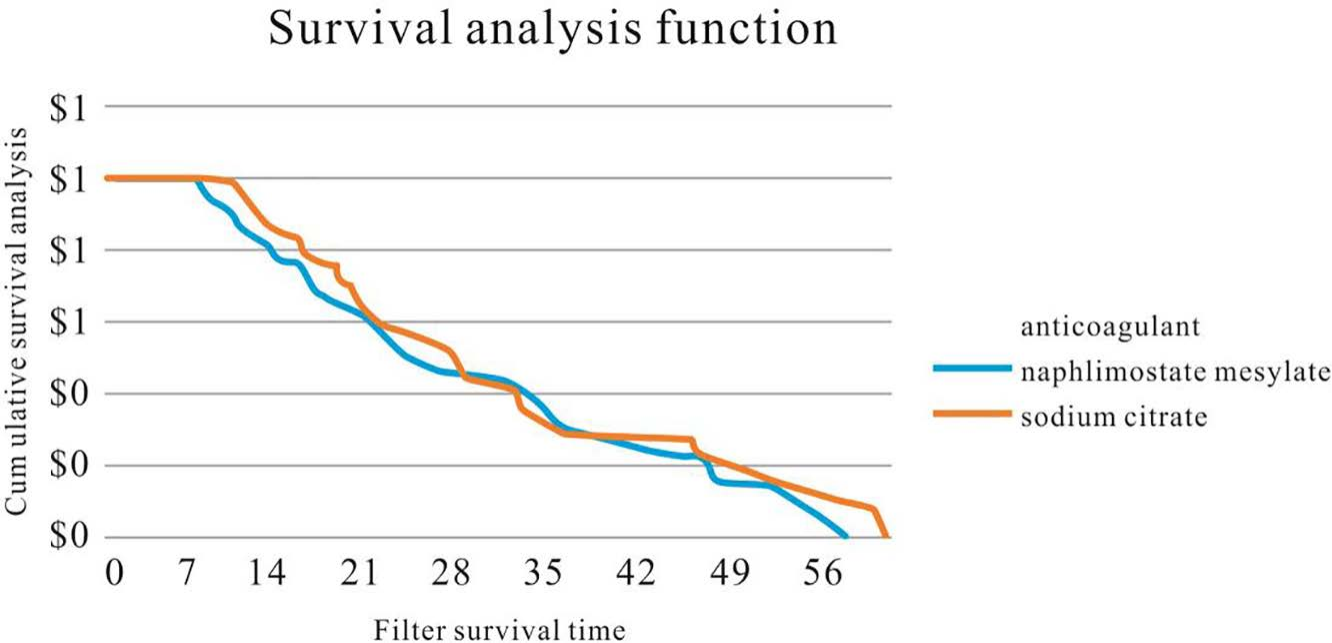
Comparison of CRRT filter life between nalmostat mesylate and sodium citrate hydrochloride group (*P* > .05). CRRT = continuous renal replacement therapy.

1. In both groups, the filter survival time was 25.5 (16.0, 43.5) vs 28.5 (18.0, 46.5) days, and the daily number of filters used was 1.16 ± 0.76 vs 1.00 ± 0.55, respectively, with no statistically significant difference (*P* > .05) observed. Table 1 shows no significant difference between the 2 groups. Figures 1 and 2 depict the box plot and Kaplan–Meier survival chart, respectively, indicating no significant difference in filter duration between the groups.
2. Pretreatment laboratory results for 39 patients in the experimental group and 36 patients in the control group showed no statistically significant differences (*P* > .05) in age, gender distribution, pre-PT, APTT, INR, hemoglobin levels, platelet count, serum potassium, serum calcium, white blood cell count, or neutrophil count (Tables 2 and 3).
3. Posttreatment laboratory findings of both groups, including PT, APTT, INR, hemoglobin levels, platelet count, serum sodium, serum potassium, serum calcium, leukocyte count, and neutrophil count, showed no statistically significant differences (*P* > .05) (Table 4).
4. Although some patients were at risk of bleeding, we did neither observe any severe bleeding events that required blood transfusion, nor did we encounter bleeding in critical organs or body cavities, and no deaths were attributed to bleeding. Regarding the assessment of adverse drug reactions, the study showed that neither the experimental nor the control group reported any severe drug-related adverse reactions during the treatment period, such as allergic reactions, granulocytopenia, or bone marrow suppression.

**Table 1:** Long survival time of filters in naphlimostat mesylate and sodium hydrochloride.

	Naphlimostat mesylate (n = 39)	Sodium citrate group (n = 36)	*P*
Filter life (h)	25.5 (16.0–43.5)	28.5 (18.0–46.5)	.15
Number of filters per day (s)	1.16 ± 0.76	1.00 ± 0.55	.21

The symbol *x̄* represents the sample mean, and *s* denotes the standard deviation. The combination of the mean and standard deviation is used to describe the central tendency and variability of the data.

**Table 2:** General conditions and disease distribution of the patients.

Primary disease [case (%)]	Naphlimostat mesylate (n = 39)	Sodium citrate group (n = 36)	*P*
Age (yr), ($\bar{x}\pm s$)	60.5 ± 14.3	63.2 ± 11.5	.432
Male/female (column)	21/18	19/17	
Hepatic failure	12 (30.7)	10 (27.7)	
Severe pneumonia	10 (25.6)	7 (19.4)	
Septic shock	17 (40.4)	19 (52.7)	

The symbol *x̄* represents the sample mean, and *s* denotes the standard deviation. The combination of the mean and standard deviation is used to describe the central tendency and variability of the data.

**Table 3:** Pretreatment laboratory findings of naphlimostat and sodium citrate hydrochloride patients ($\bar{x}\pm \;s$, s).

	Naphlimostat mesylate (n = 39)	Sodium citrate group (n = 36)	*t*	*P*
PT ($\bar{x}\pm s$) (s)	28.60 ± 11.95	22.59 ± 15.60	0.866	.409
APTT ($\bar{x}\pm s$) (s)	55.65 ± 20.82	50.32 ± 10.34	0.279	.786
INR ($\bar{x}\pm s$) (s)	2.29 ± 0.29	4.23 ± 2.56	−0.727	.484
Hemoglobin ($\bar{x}\pm s$) (g/L)	80.30 ± 6.19	99.90 ± 10.62	−1.762	.112
Platelet count ($\bar{x}\pm s$) (×10^9^/L)	57.57 ± 20.75	96.00 ± 25.39	−1.006	.341
Serum sodium ($\bar{x}\pm s$) (mmol/L)	136.36 ± 1.79	143.91 ± 2.31	−2.364	.05
Serum potassium ($\bar{x}\pm s$) (mmol/L)	4.40 ± 0.31	4.50 ± 0.43	−0.186	.856
Serum calcium ($\bar{x}\pm s$) (mmol/L)	1.10 ± 0.05	0.91 ± 0.11	1.785	.104
White blood cell count ($\bar{x}\pm s$) (×10^9^/L)	9.48 ± 1.22	13.96 ± 2.64	−1.411	.192

The symbol *x̄* represents the sample mean, and *s* denotes the standard deviation. The combination of the mean and standard deviation is used to describe the central tendency and variability of the data.

APTT = activated partial thromboplastin time, INR = international normalized ratio.

**Table 4:** General conditions of patients and pretreatment laboratory findings ($\bar{x}\pm \;s$, s).

	Naphlimostat mesylate group	Sodium citrate group	Mean difference (95% CI)	*t*	*P*
PT ($\bar{x}\pm s$) (s)	35.95 ± 33.14	23.71 ± 19.37	12.24 (−14.11 to 38.59)	1.051	.321
APTT ($\bar{x}\pm s$) (s)	60.48 ± 24.07	47.44 ± 29.90	13.04 (−18.25 to 44.33)	0.943	.37
INR ($\bar{x}\pm s$) (s)	3.05 ± 2.83	4.91 ± 10.60	−1.86 (−10.06 to 6.33)	−0.515	.619
Hemoglobin ($\bar{x}\pm s$) (g/L)	82.10 ± 15.01	93.10 ± 26.86	−11.00 (−32.30 to 10.30)	−1.168	.273
Platelet count ($\bar{x}\pm s$) (×10^9^/L)	66.70 ± 59.87	70.10 ± 48.85	−3.40 (−61.41 to 54.61)	−0.133	.897
Serum sodium ($\bar{x}\pm s$) (mmol/L)	138.80 ± 5.92	142.00 ± 3.59	−3.20 (−7.89 to 1.49)	−1.542	.157
Serum potassium ($\bar{x}\pm s$) (mmol/L)	4.00 ± 0.52	3.96 ± 0.61	0.04 (−0.57 to 0.65)	0.147	.886
Serum calcium ($\bar{x}\pm s$) (mmol/L)	1.13 ± 0.19	0.99 ± 0.28	0.14 (−0.05 to 0.33)	1.659	.128
White blood cell count ($\bar{x}\pm s$) (×10^9^/L)	10.82 ± 5.81	12.54 ± 6.32	−1.72 (−7.41 to 3.96)	−0.686	.51
Neutrophils ($\bar{x}\pm s$) (×10^9^/L)	8.73 ± 5.05	10.89 ± 5.94	−2.16 (−7.34 to 3.03)	−0.941	.371

The symbol *x̄* represents the sample mean, and *s* denotes the standard deviation. The combination of the mean and standard deviation is used to describe the central tendency and variability of the data.

APTT = activated partial thromboplastin time, INR = international normalized ratio.

## 4. Discussion

The use of appropriate anticoagulants is crucial to prolong filter lifespan and minimize bleeding complications during treatment. Heparin is currently the standard anticoagulant used during CRRT^[14,15]^; however, it poses an increased bleeding risk for patients with coagulation dysfunction, potentially affecting treatment efficacy and prognosis. In cases where CRRT is necessary alongside AKI and coagulation issues, sodium citrate is recommended as the preferred anticoagulant for high-risk bleeding patients.^[16,17]^ Nonetheless, due to its chelation of calcium ions, sodium citrate can lead to hypocalcemia and related complications,^[18,19]^ necessitating caution in patients with severe multiorgan failure and persistent hyperlactatemia.^[20,21]^ Regular electrolyte monitoring, while essential, not only raises treatment costs but also adds to medical staff workload.

A 7-year retrospective study compared the filter lifespan of 3 common anticoagulants. The results revealed that 279 patients received heparin treatment (56.60%), with the filter’s median lifespan being 14.08 hours (7.30, 21.50). In 128 patients treated with NM (26.00%), the median lifespan of the filter was 16.42 hours (10.49, 22.76). Additionally, 86 patients received sodium citrate treatment (17.40%), with the filter’s median lifespan being 31.06 hours (19.25, 48.75).^[22]^ However, it is important to note that these findings were derived from patients with severe burns, which may not fully align with the demographics of our study. Another study comparing sodium citrate and NM found no significant difference in filter lifespan.^[23]^ Additionally, a 2013 study from the University of Tokyo indicated that NM could achieve a median filter survival of over 40 hours, suggesting comparable efficacy to other anticoagulants in terms of filter longevity.^[24]^ Our own assessment of filter lifespan also showed NM slightly outperforming the sodium citrate group (34.3 ± 4.5 vs 33.0 ± 3.9 hours), though the difference was not statistically significant (*P* > .05).

Studies have demonstrated that NM effectively reduces hemorrhagic complications in severe bleeding patients compared with heparin, lowering the incidence from 64% to 4%.^[25]^ NM, a potent serine protease inhibitor, exhibits rapid blood elimination with a short half-life of only 8 minutes, making it an excellent in vitro anticoagulant. Despite potential side effects such as granulocytosis, hyperkalemia, and allergic reactions, these occurrences are rare.^[10]^ The combination of strong anticoagulant properties and low side effect incidence suggests that NM may be particularly suitable for patients with coagulopathy and AKI.^[26]^ In our study, we compared changes in coagulation factors (APTT, PT, INR) between the experimental group (NM) and a control group (sodium citrate). We found no significant differences in the magnitude of these changes. Additionally, our analysis of serum sodium, potassium, calcium levels, and inflammatory markers before and after treatment showed no significant deviations, indicating no electrolyte imbalances or abnormal increases in inflammatory indicators. This stability is primarily attributed to the very short half-life of NM (8 minutes), which acts exclusively within the extracorporeal circuit. Finally, we did not observe any bleeding events or severe adverse reactions. Furthermore, upon cessation of CRRT in the NM group, we observed increases in hemoglobin and platelet counts compared with pre-CRRT levels. This differs from the sodium citrate control group, where sodium citrate dosing requires careful monitoring (typically within a narrow range of 4–6 mmol/L) to avoid complications such as alkalosis, acidosis, hyponatremia, and hypernatremia,^[27]^ necessitating frequent blood draws for monitoring. In contrast, NM operates through different mechanisms, reducing the need for frequent blood draws.

### 4.1. Conclusion

NM does not increase the risk of bleeding in coagulopathy patients undergoing CRRT, while maintaining filter survival time, establishing its safety and effectiveness as an in vitro anticoagulant.

### 4.2. Limitations

It is a single-center study; the sample size is too small to allow for further subgroup analysis. Future research should prioritize prospective randomized controlled trials involving multiple centers and larger sample sizes to further investigate its potential.

## Author contributions

**Conceptualization:** Chun Liu.

**Data curation:** Chun Liu.

**Formal analysis:** Chun Liu.

**Investigation:** Binglin Song.

**Methodology:** Binglin Song.

**Project administration:** Binglin Song.

**Validation:** Kangrui Fu.

**Visualization:** Kangrui Fu.

**Writing – original draft:** Kangrui Fu.

## References

[1] Peters E Antonelli M Wittebole X. A worldwide multicentre evaluation of the influence of deterioration or improvement of acute kidney injury on clinical outcome in critically ill patients with and without sepsis at ICU admission: results from The Intensive Care Over Nations audit. Crit Care. 2018;22:1–11. 30075798 10.1186/s13054-018-2112-z PMC6091052

[2] Kellum JA Prowle JR. Paradigms of acute kidney injury in the intensive care setting. Nat Rev Nephrol. 2018;14:217–30. 29355173 10.1038/nrneph.2017.184

[3] Cronin RE Reilly RF. Unfractionated heparin for hemodialysis: still the best option. Semin Dial. 2010;23:510–15. 21039876 10.1111/j.1525-139X.2010.00770.x PMC3229102

[4] Shen JI Winkelmayer WC. Use and safety of unfractionated heparin for anticoagulation during maintenance hemodialysis. Am J Kidney Dis. 2012;60:473–86. 22560830 10.1053/j.ajkd.2012.03.017 PMC4088960

[5] Hetzel GR Sucker C. The heparins: all a nephrologist should know. Nephrol Dial Transplant. 2005;20:2036–42. 16030035 10.1093/ndt/gfi004

[6] Lee GM Arepally GM. Heparin-induced thrombocytopenia. Hematology Am Soc Hematol Educ Program. 2013;2013:668–74. 24319250 10.1182/asheducation-2013.1.668 PMC4153428

[7] Legrand M Tolwani A. Anticoagulation strategies in continuous renal replacement therapy. Semin Dial. 2021;34:416–22. 33684244 10.1111/sdi.12959

[8] Kidney Disease: Improving Global Outcomes (KDIGO) Acute Kidney Injury Work Group. KDIGO clinical practice guidelines for acute kidney injury. Kidney Int Suppl. 2012;2:1–138.

[9] Arimura T Abe M Shiga H Katayama H Kaizu K Oda S. Clinical study of blood purification therapy in critical care in Japan: results from the survey research of the Japan Society for Blood Purification in Critical Care in 2013. J Artif Organs. 2017;20:244–51. 28600615 10.1007/s10047-017-0968-3

[10] Hwang SD Hyun YK Moon SJ Lee SC Yoon SY. Nafamostat mesilate for anticoagulation in continuous renal replacement therapy. Int J Artif Organs. 2013;36:208–16. 23404639 10.5301/IJAO.5000191

[11] Lee YK Lee HW Choi KH Kim BS. Ability of nafamostat mesilate to prolong filter patency during continuous renal replacement therapy in patients at high risk of bleeding: a randomized controlled study. PLoS One. 2014;9:e108737. 25302581 10.1371/journal.pone.0108737 PMC4193755

[12] Song J-C Yang L-K Zhao W. Chinese expert consensus on diagnosis and treatment of trauma-induced hypercoagulopathy. Mil Med Res. 2021;8:25. 33840386 10.1186/s40779-021-00317-4 PMC8040221

[13] Jörres A. Intensivmedizin, notfallmedizin, schmerztherapie: AINS, Evidence-based renal replacement therapy – intermittent versus CRRT. Anasthesiol Intensivmed Notfallmed Schmerzther. 2013;48:108–13. 23504467 10.1055/s-0033-1336587

[14] Tandukar S Palevsky PM. Continuous renal replacement therapy: who, when, why, and how. Chest. 2019;155:626–38. 30266628 10.1016/j.chest.2018.09.004 PMC6435902

[15] Ronco C Reis T. Continuous renal replacement therapy and extended indications. Semin Dial. 2021:550–60. 33711166 10.1111/sdi.12963

[16] Thompson A Li F Gross AK. Considerations for medication management and anticoagulation during continuous renal replacement therapy. AACN Adv Crit Care. 2017;28:51–63. 28254856 10.4037/aacnacc2017386

[17] Annich GM Zaulan O Neufeld M Wagner D Reynolds MM. Thromboprophylaxis in extracorporeal circuits: current pharmacological strategies and future directions. Am J Cardiovasc Drugs. 2017;17:425–39. 28536932 10.1007/s40256-017-0229-0

[18] Liu C Mao Z Kang H Hu J Zhou F. Regional citrate versus heparin anticoagulation for continuous renal replacement therapy in critically ill patients: a meta-analysis with trial sequential analysis of randomized controlled trials. Crit Care. 2016;20:1–13. 27176622 10.1186/s13054-016-1299-0 PMC4866420

[19] Kalb R Kram R Morgera S Slowinski T Kindgen‐Milles D. Regional citrate anticoagulation for high volume continuous venovenous hemodialysis in surgical patients with high bleeding risk. Ther Apher Dial. 2013;17:202–12. 23551677 10.1111/j.1744-9987.2012.01101.x

[20] Bai M Zhou M He L. Citrate versus heparin anticoagulation for continuous renal replacement therapy: an updated meta-analysis of RCTs. Intensive Care Med. 2015;41:2098–110. 26482411 10.1007/s00134-015-4099-0

[21] Wu M-Y Hsu Y-H Bai C-H Lin Y-F Wu C-H Tam K-W. Regional citrate versus heparin anticoagulation for continuous renal replacement therapy: a meta-analysis of randomized controlled trials. Am J Kidney Dis. 2012;59:810–8. 22226564 10.1053/j.ajkd.2011.11.030

[22] Yue Q Wu H Xi M Li F Li T Li Y. Filter lifespan, treatment effect, and influencing factors of continuous renal replacement therapy for severe burn patients. J Burn Care Res. 2024;45:764–70. 38113522 10.1093/jbcr/irad196 PMC11073580

[23] Liu D Zhao J Xia H. Nafamostat mesylate versus regional citrate anticoagulation for continuous renal replacement therapy in patients at high risk of bleeding: a retrospective single-center study. Eur J Med Res. 2024;29:72. 38245802 10.1186/s40001-024-01660-7 PMC10799389

[24] Maruyama Y Yoshida H Uchino S. Nafamostat mesilate as an anticoagulant during continuous veno-venous hemodialysis: a three-year retrospective cohort study. Int J Artif Organs. 2011;34:571–6. 21786254 10.5301/IJAO.2011.8535

[25] Ohtake Y Hirasawa H Sugai T. Nafamostat mesylate as anticoagulant in continuous hemofiltration and continuous hemodiafiltration. Contrib Nephrol. 1991;93:215–7. 1666354 10.1159/000420222

[26] Doi K Nishida O Shigematsu T. The Japanese clinical practice guideline for acute kidney injury 2016. Clin Exp Nephrol. 2018;22:985–1045. 30039479 10.1007/s10157-018-1600-4 PMC6154171

[27] Lanckohr C Hahnenkamp K Boschin M. Continuous renal replacement therapy with regional citrate anticoagulation: do we really know the details? Curr Opin Anaesthesiol. 2013;26:428–37. 23673990 10.1097/ACO.0b013e3283620224

